# Access to and price trends of antidiabetic, antihypertensive, and antilipidemic drugs in outpatient settings of the Universal Coverage Scheme in Thailand

**DOI:** 10.1371/journal.pone.0211759

**Published:** 2019-02-20

**Authors:** Chulaporn Limwattananon, Onanong Waleekhachonloet

**Affiliations:** 1 Division of Clinical Pharmacy, Faculty of Pharmaceutical Sciences, Khon Kaen University, Khon Kaen, Thailand; 2 Department of Clinical Pharmacy, Faculty of Pharmacy, Mahasarakham University, Mahasarakham, Thailand; Tulane University, UNITED STATES

## Abstract

Under the Universal Coverage Scheme (UCS) with payment per capita for outpatient (OP) services, hospitals’ financial risks will rise if access to essential drugs increases. This study examined trends in access to and price of essential drugs for noncommunicable diseases (NCDs) and an overall purchasing price index (PPI) for an OP drug basket from public hospitals. To examine drug access, OP prescription data from 2010–2012 were obtained from the UCS. Access to thirteen drugs for diabetes, hypertension, and dyslipidemia was examined for trend using a time-series analysis. To calculate the PPI, drugs in the same dataset in 2010 that each contributed at least 0.2% of the total OP drug expenditure (N = 118 items) were selected together with drugs expected for near future growth (N = 48 items). The PPI was constructed from purchasing prices in 16 hospitals using a standard method developed by the International Labour Organization. Based on 166 drug items accounting for 75% of OP drug expenditures, the overall PPI continually declined by 6.8% from 2010 to 2012. Access to the 13 selected NCD drugs, accounting for 22% of the total OP drug expenditure increased from 22 to 30 per 1,000 population for antidiabetics, 27 to 47 for antihypertensive agents, and 32 to 53 for antilipidemics from 2010–2012. Growth in the study drug recipients was relatively higher than that in the population and diagnosed patients. Due to generic market competition, metformin, glipizide, amlodipine, losartan, simvastatin, atorvastatin, and fenofibrate prices decreased by 6–22%. Antiretrovirals and risperidone prices decreased by more than 10% due to price negotiation by the UCS. Access to essential drugs for diabetes, hypertension and dyslipidemia has increased. A decline in the PPI could contain essential drug expenditure when the demand for the drugs increased. Generic market competition and price negotiation by the UCS led to price reduction.

## Introduction

Thailand has achieved universal health coverage (UHC) since introduction of the Universal Coverage Scheme (UCS) for the uninsured and medical indigents in 2002.[1] Financed by general government revenue, the UCS relies mostly on public hospitals to provide inpatient (IP) and outpatient (OP) care to approximately 47 million members, covering three-quarters of the Thai population. Each year, the National Health Security Office (NHSO), which is the administrative body of the UCS, establishes payment contracts with hospitals for IP services based on global budgeting and diagnosis-related groups (DRGs). The hospital OP costs, which are largely driven by drug utilization, are compensated based on the fixed annual per capita payment rate.[2]

During the first decade (2003–2013) of UHC, the per capita UCS budget in total grew from 35.4 US$ to 78.8 US$.[3] However, the OP budget share decreased by 29.4% and the number of OP visits per capita increased by 27.3% over the same period.[3] Increasing demand for healthcare would pressure hospitals not only for services but also in terms of the cost burden. For OP medical services, the financial risk of hospitals would rise if the increase in drug prices was disproportionately higher than that of the capitation payment. Given the fixed OP payment rate across the board, tracking utilization is essential as a safeguard for adequate care. Monitoring the provider’s acquisition of resources and the major components of expenditures are essential to secure adequate budget allocation from the NHSO.[4]

Global communities have advocated access to essential medicines for noncommunicable diseases (NCDs).[5] Medical treatments for diabetes, hypertension, and dyslipidemia are free of charge for the UCS population. Hospital admissions for patients with diabetes and hypertension have exhibited an increasing trend.[6] As ambulatory care sensitive conditions, both diabetes and hypertension can be effectively controlled through drug treatment in an OP setting.[7,8] Whether the increasing hospitalization for diabetes and hypertension has root causes from access to screening, diagnosis and treatment has not been examined. Monitoring the use of antidiabetic, antihypertensive and antilipidemic drugs will help shed light on how well the UCS population obtains access to essential treatments for these NCDs.

Monitoring the price trends of drugs helps predict whether the UCS budget allocated for hospitals can offset costs in cases of an increase in access to essential treatments for common NCDs. To be comparable with other health resources, drug price monitoring should account for the whole streams of major medical treatments. This process requires a summary and normalized measure of price changes, which is known as the price index, for a reasonable basket of drugs prescribed for UCS members.

This study examined trends in the access to and prices of essential medicines for diabetes, hypertension and dyslipidemia in OP services at the end of the first UHC decade (fiscal year, FY 2010–2012). In addition, the purchasing price index (PPI) was constructed as a summary measure for OP drugs purchased by public hospitals using standard methodology. The findings will hint at whether hospitals can bear the costs of drugs when demand for medication increases.

## Materials and methods

### Drug access

Variables for the drug access analysis were derived in four levels, including (1) the population in the study hospital areas, (2) patients who visited the study hospitals with a diagnosis of diabetes (the International Classification of Diseases–ICD-10^th^ revision codes, E10-E14) and cardiovascular system diseases (CVS; ICD-10 codes, I10-I99), (3) patients prescribed antidiabetic, antihypertensive and antihyperlipidemic drugs, and (4) the quantity of the prescribed medicines, except for injectable insulin, as measured by the World Health Organization (WHO) defined daily doses (DDDs).

#### Data source

The OP prescription data at the individual patient level and the UCS population data were provided by the NHSO. The datasets covered services for UCS members during FY 2010–2012 in 12 provincial hospitals and 75 district hospitals with a total of 10,320 beds. These hospitals were the main contracting providers in 8 of 76 provinces across the country and covered approximately 10% of the Thai population. Variables for each patient consisted of encrypted personal identification, date of birth, hospital identification, ICD-10 diagnosis, dates of hospital visits, prescribed medicines as identified by the Anatomical Therapeutic Chemical (ATC) classification, and quantities and dates of prescriptions. The ATC codes, such as “A10BA02” have five levels. For ATC level 1, drugs are divided into 14 main groups according to the organ or system they act upon and are represented by one alphabetical letter. ATC level 2 consists of 2 digits indicating the major therapeutic class. ATC level 3 has one letter indicating a pharmacological subgroup. ATC level 4 has one letter indicating a chemical subgroup. Lastly, ATC level 5 has two digits indicating an individual chemical substance, which is the generic name equivalent.[9]

#### Drug selection

Drugs used for diabetes, hypertension and dyslipidemia were shortlisted for access and price monitoring, because these three NCDs had high priority for disease control.[10] The ATC codes of these drugs at level 2 are A10 for antidiabetics, C08 (calcium channel blockers) plus C09 (agents acting on the renin-angiotensin system) for antihypertensive agents, and C10 for antilipidemic drugs. Diuretics (ATC code, C03) used for hypertension were not selected, because those drugs deemed essential were inexpensive and had been widely prescribed for a long time. The drugs that appear in Thailand’s National List of Essential Medicines (NLEM) are fully covered by the UCS and hence have been selected for this study.[11] The generic entities (level 5 ATC) of the essential drugs are insulin, metformin, glibenclamide, glipizide and pioglitazone for A10, amlodipine and nifedipine for C08, enalapril and losartan for C09, and simvastatin, atorvastatin, gemfibrozil and fenofibrate for C10.

#### Data analysis

The analysis covered UCS members aged 15 years and over. Access to each selected drug was measured in terms of population coverage, which was a ratio between the number of drug recipients and the total population in the catchment areas of the study hospitals in a given year. The quarterly trend in access was estimated using a time-series analysis. To account for serial correlation between observations in adjacent quarters, a generalized least square that accounted for the first-order autoregression was employed using Prais-Winsten transformation.[12]

To disentangle overall trends in access by population, the following components were decomposed and estimated for an annual change. First, demographic factors were broken down with respect to adult and elderly populations. Second, epidemiological factors were examined, using the ratios of patients diagnosed with diabetes or CVS diseases to the total population residing in the hospital areas. Third, access was additionally determined by the ratio of drug recipients to the diagnosed patients. Lastly, drug use quantity was estimated using the DDDs per recipient for each drug group.

### Price change and price index

For each of the 13 drugs selected for access measurement, price changes were calculated based on the geometric mean price of every quarter (Q) relative to that of Q1, FY 2010.

Conceptually, the price index represents a relative change in a price ratio between the current period and the base period (set at 100% in FY 2010), and accounts for weighted expenditures. Price indices were calculated at ATC levels 5 and 1 and as overall for drugs selected into a basket. The overall price index is a summary scale that measures the weighted averages of price changes of individual drugs using an aggregate statistic. Monitoring of the overall price index was recommended by Health Action International and the WHO.[13] In this study, the PPI was calculated quarterly during FY 2010–2012. The method used to derive the overall price index conformed to the standard method developed by the International Labour Organization.[14] The detailed calculation steps with formula are summarized in the supplementary information (S1 File).

#### Data source

The dataset for development of the drug basket was OP prescriptions in FY 2010, which was used for the access measurement. Data on unit prices were obtained voluntarily from 16 public hospitals located in all geographic regions of Thailand. The obtained data were the values of net prices specific to the dosage forms, strengths and packages of the drug items purchased by the hospitals in each quarter from various vendors during FY 2010–2012.

#### Drug selection

Selection of a particular drug into a meaningful basket for the price index was based on the contribution to the total OP drug expenditure. In the abovementioned UCS dataset for the access measure, 118 drugs identified at ATC level 5, in which each drug contributed to at least 0.2% of the total OP drug expenditure in the base year (FY 2010), were included in the basket. Additionally, 48 drugs with expected near-future growth were selected. For each drug, dosage forms that accounted for at least 60% of each drug’s expenditure were selected into the basket. Of 14 main groups at ATC level 1, 3 groups, including “D” (dermatologicals), “P” (antiparasitic products), and “V” (various products), were not selected for the basket. As a result, the 11 selected groups at ATC level 1, each containing 3–31 drug items at ATC level 5 constituted the basket of 166 drugs in total. For the 166 selected drugs, the shared expenditure (a multiplication between price and volume) of each drug was summed to 100%, and this individual share was referred to as the expenditure contribution.

#### Data analysis

For every quarter, a geometric mean for an individual drug at ATC level 5 across dosage forms and strengths in the 16 hospitals that provided purchasing price data was calculated for a unit price. For each drug in the base year (FY 2010), the ratio of the unit price at each Q to the average of the unit prices across Q1-Q4 of the base year was weighted by expenditure contribution to obtain the weighted quarterly price ratio. An item price index at ATC level 5 for Q1-Q4 in the base year was calculated by dividing the weighted price ratio by the expenditure contribution (in percentages). For Q5 onwards, the item price index in each Q was calculated using the Lowe index and modified Laspeyres index formula which are chain-base methods.[13]

To calculate the price index at ATC level 1 for every Q, the weighted price ratios at ATC level 5 were summed. In each of the first four quarters (Q1-Q4), the price index at ATC level 1 is the ratio between the summed weighted ratios across ATC level 5 and the summed expenditure contribution at ATC level 1. From Q5 onwards, the aggregate index at ATC level 1 in a current quarter was chained to the index from a previous adjacent Q.

The overall price index for the highest aggregate (i.e., the whole basket of 166 drugs) was calculated using the same method by summing the weighted ratios for the base-year quarters; then, the summed expenditure contribution was equal to 100 and chained to the previous quarter index for Q5 onwards.

The overall price index was recalculated for a subset of the basket after excluding 20 drugs for which UCS implemented central purchasing via price negotiation by volume-based agreement. These drugs included drugs for the treatment of HIV/AIDS, selected cancers, and chronic kidney disease.

## Results

### Drug access

**Fig 1:**
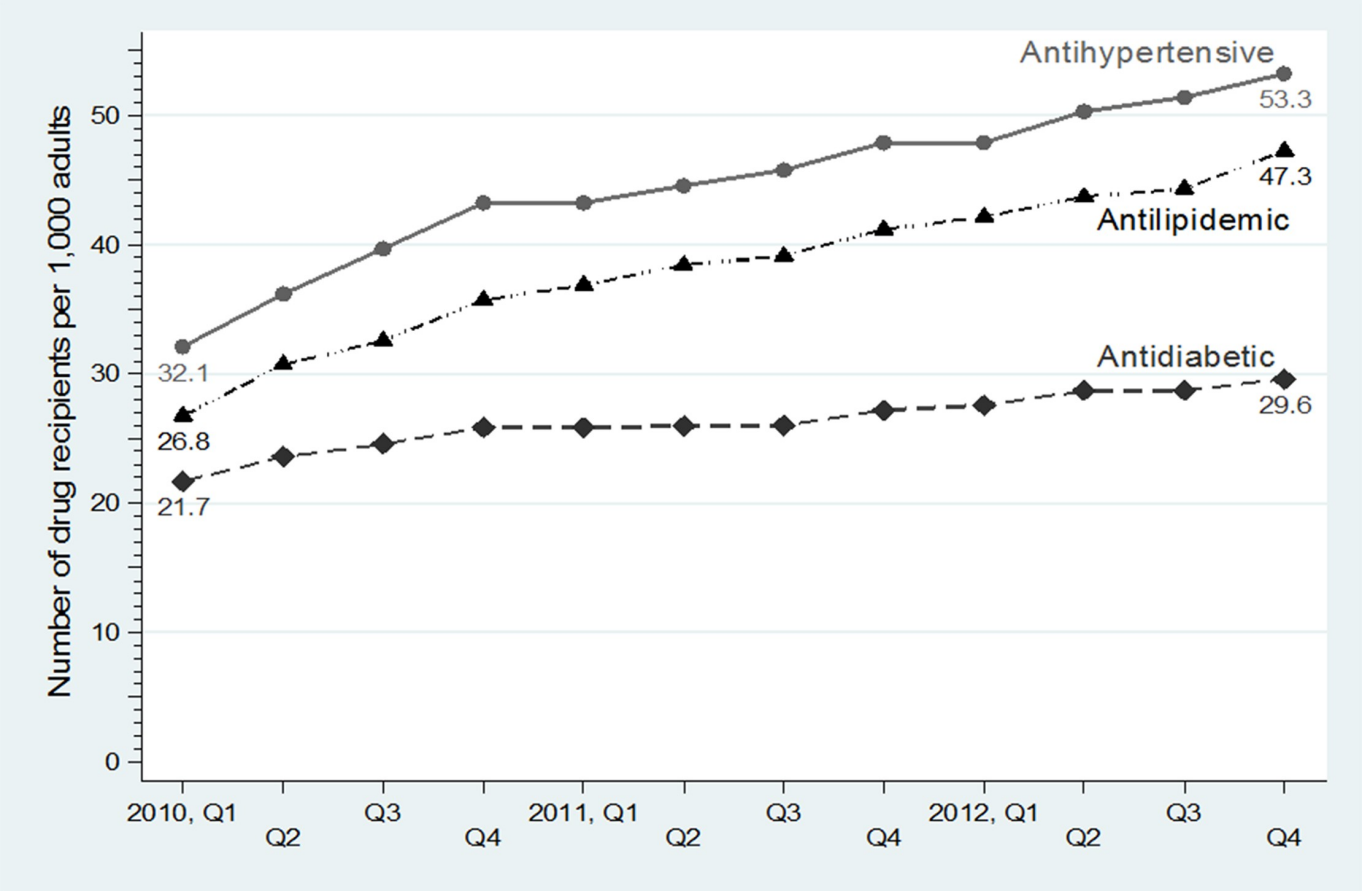
Trends in access to antidiabetic, antihypertensive and antilipidemic drugs in the NLEM per 1,000 population*, Q1, FY 2010—Q4, FY 2012. *Age 15 years or older. NLEM, National List of Essential Medicines; Q, quarter; FY, fiscal year. Antidiabetics: insulin, metformin, glibenclamide, glipizide, and pioglitazone; Antihypertensive agents: amlodipine, nifedipine, enalapril, and losartan; Antilipidemics: simvastatin, atorvastatin, gemfibrozil, and fenofibrate.

The increasing trend in access to essential drugs for diabetes, hypertension and dyslipidemia for the UCS population is illustrated in Fig 1 and Table 1. From Q1, FY 2010 to Q4, FY 2012, the number per 1,000 population of patients receiving antidiabetics grew gradually from 22 to 30, whereas those for antihypertensive agents and antilipidemics grew rapidly from 32 to 53 and from 27 to 47, respectively.

**Table 1:** Expenditure on and access to antidiabetic, antihypertensive and antilipidemic drugs, FY 2010–2012.

Drug	% share of OP drug expenditure (FY 2010)	Quarterly change in drug recipients per 1,000 population*	P-value	No. of drug recipients per 1,000 population					
				2010 Q1	2010 Q3	2011 Q1	2011 Q3	2012 Q1	2012 Q3
Antidiabetics									
Insulin	3.0%	0.27	<0.0001	3.58	4.35	4.76	5.39	5.70	6.27
Metformin	4.0%	0.53	<0.0001	15.81	18.29	19.31	19.39	20.57	21.37
Glibenclamide	1.3%	-0.20	<0.0001	11.08	10.46	10.38	9.43	9.59	9.15
Glipizide	1.2%	0.55	<0.0001	4.03	5.78	6.77	7.51	8.42	9.54
Pioglitazone	0.6%	0.14	<0.0001	0.75	1.16	1.46	1.67	1.86	2.08
Overall	10.1%	0.63	<0.0001	21.70	24.58	25.88	26.06	27.58	28.75
Antihypertensive agents									
Amlodipine	2.5%	1.32	<0.0001	14.39	18.87	21.21	23.69	25.34	28.07
Nifedipine	0.6%	-0.04	0.0443	1.91	2.21	1.95	1.81	1.78	1.70
Enalapril	2.3%	0.81	0.0002	20.52	24.54	26.40	27.03	27.72	29.31
Losartan	0.5%	0.46	<0.0001	1.56	2.54	3.40	4.37	5.11	6.17
Overall	5.9%	1.79	<0.0001	32.11	39.69	43.27	45.76	47.91	51.43
Antilipidemics									
Simvastatin	2.6%	1.51	<0.0001	20.84	25.39	29.02	31.54	33.67	35.73
Atorvastatin	2.0%	0.03	0.0063	0.07	0.10	0.09	0.14	0.16	0.33
Gemfibrozil	1.2%	0.26	0.001	6.87	8.36	9.27	8.80	9.69	9.51
Fenofibrate	0.2%	0.04	0.0004	0.20	0.34	0.46	0.51	0.54	0.56
Overall	6.0%	1.71	<0.0001	26.78	32.58	36.87	39.14	42.17	44.34

FY, fiscal year; OP, outpatient; Q, quarter

* based on time-series analysis of 12 quarters from Q1, FY 2010 to Q4, FY 2012

Of the 13 study drugs that accounted for 22% of the total OP drug expenditure, the top four were metformin, amlodipine, enalapril and simvastatin, which shared 52% (Table 1). From Q1, FY 2010 to Q4, FY 2012, the number per 1,000 population of patients receiving metformin increased from 16 to 21, amlodipine from 14 to 28, enalapril from 21 to 29, and simvastatin from 21 to 36. On average, the quarterly growth rate over the same period for each of the top four drugs ranged from 0.53 to 1.51 per 1,000 population. Growth in access to the rest of study drugs was less than 1 per 1,000 population. Use of nifedipine was relatively stable, whereas that of glibenclamide showed a downward trend in FY 2012 despite being comparable to glipizide.

The mean ages of the antidiabetics, antihypertensive agents and antilipidemic receipients in FY 2010 were 59, 62 and 61 years, respectively (S1 Table). The mean ages and the elderly proportion of drug recipients increased over the three-year period.

During FY 2010–2012, the numbers of the UCS population who were adults or elderly and the elderly alone increased annually by 0.9 and 3.5%, respectively (Table 2). Per 1,000 population, the average annual growth in patients diagnosed with diabetes and CVS diseases was 3.1 and 5.0%, respectively. Growth in the recipients of the study drugs was relatively higher than that in the population and the diagnosed patients. For antidiabetics, the average annual growth was 8.1 per 1,000 population but was 4.7 per 100 patients with diabetes. For antihypertensive agents, the average growth was 15.7 per 1,000 population but was 9.7 per 100 patients with CVS diseases. For antilipidemics, the average growth was 20.2 per 1,000 population but was 13.8 per 100 patients with CVS diseases. The DDDs per recipient of oral antidiabetics and antihypertensive agents in each year were higher than those for one-year duration of use, because some patients received drugs for more than one item per visit (S1 Table). However, the DDDs for all three drug classes did not show an increasing trend.

**Table 2:** Population, patients, drug recipients, defined daily doses, and annual changes, FY 2010–2012.

	Number			Annual change (%)		
	2010	2011	2012	Year to year		Average
				2011	2012	
Population						
All age groups	3,493,783	3,505,251	3,517,835	0.3%	0.4%	0.3%
Adults and elderly (≥ 15 years)	2,675,651	2,695,613	2,723,356	0.7%	1.0%	0.9%
Elderly (≥ 60 years)	501,128	516,310	536,182	3.0%	3.8%	3.5%
Patients ≥ 15 years						
Diabetes	117,307	121,425	126,859	3.5%	4.5%	4.1%
Cardiovascular diseases	294,751	311,685	329,859	5.7%	5.8%	6.0%
Patients per 1,000 population						
Diabetes	43.8	45.0	46.6	2.7%	3.4%	3.1%
Cardiovascular diseases	110.2	115.6	121.1	5.0%	4.8%	5.0%
Drug recipients						
Antidiabetics	81,978	90,558	96,938	10.5%	7.0%	9.1%
Antidiabetics, oral	73,766	81,586	87,200	10.6%	6.9%	9.1%
Antihypertensive agents	139,311	168,485	186,279	20.9%	10.6%	16.9%
Antilipidemics	119,193	148,578	170,281	24.7%	14.6%	21.4%
Drug recipients per 1,000 population						
Antidiabetics	30.6	33.6	35.6	9.6%	6.0%	8.1%
Antidiabetics, oral	27.6	30.3	32.0	9.8%	5.8%	8.1%
Antihypertensive agents	52.1	62.5	68.4	20.0%	9.4%	15.7%
Antilipidemics	44.5	55.1	62.5	23.7%	13.4%	20.2%
Drug recipients per 100 patients						
Antidiabetics	69.9	74.6	76.4	6.7%	2.5%	4.7%
Antidiabetics, oral	62.9	67.2	68.7	6.9%	2.3%	4.7%
Antihypertensive agents	47.3	54.1	56.5	14.4%	4.5%	9.7%
Antilipidemics	40.4	47.7	51.6	17.9%	8.3%	13.8%
Total defined daily doses (DDDs)						
Antidiabetics, oral	30,812,616	31,491,306	34,798,400	2.2%	10.5%	6.5%
Antihypertensive agents	68,072,912	71,763,792	81,034,536	5.4%	12.9%	9.5%
Antilipidemics	21,945,368	23,592,838	25,791,900	7.5%	9.3%	8.8%
DDDs per drug recipient						
Antidiabetics, oral	417.7	386.0	399.1	-7.6%	3.4%	-2.2%
Antihypertensive agents	488.6	425.9	435.0	-12.8%	2.1%	-5.5%
Antilipidemics	184.1	158.8	151.5	-13.8%	-4.6%	-8.9%

FY, fiscal year

### Price trends of antidiabetic, antihypertensive and antilipidemic drugs

**Fig 2:**
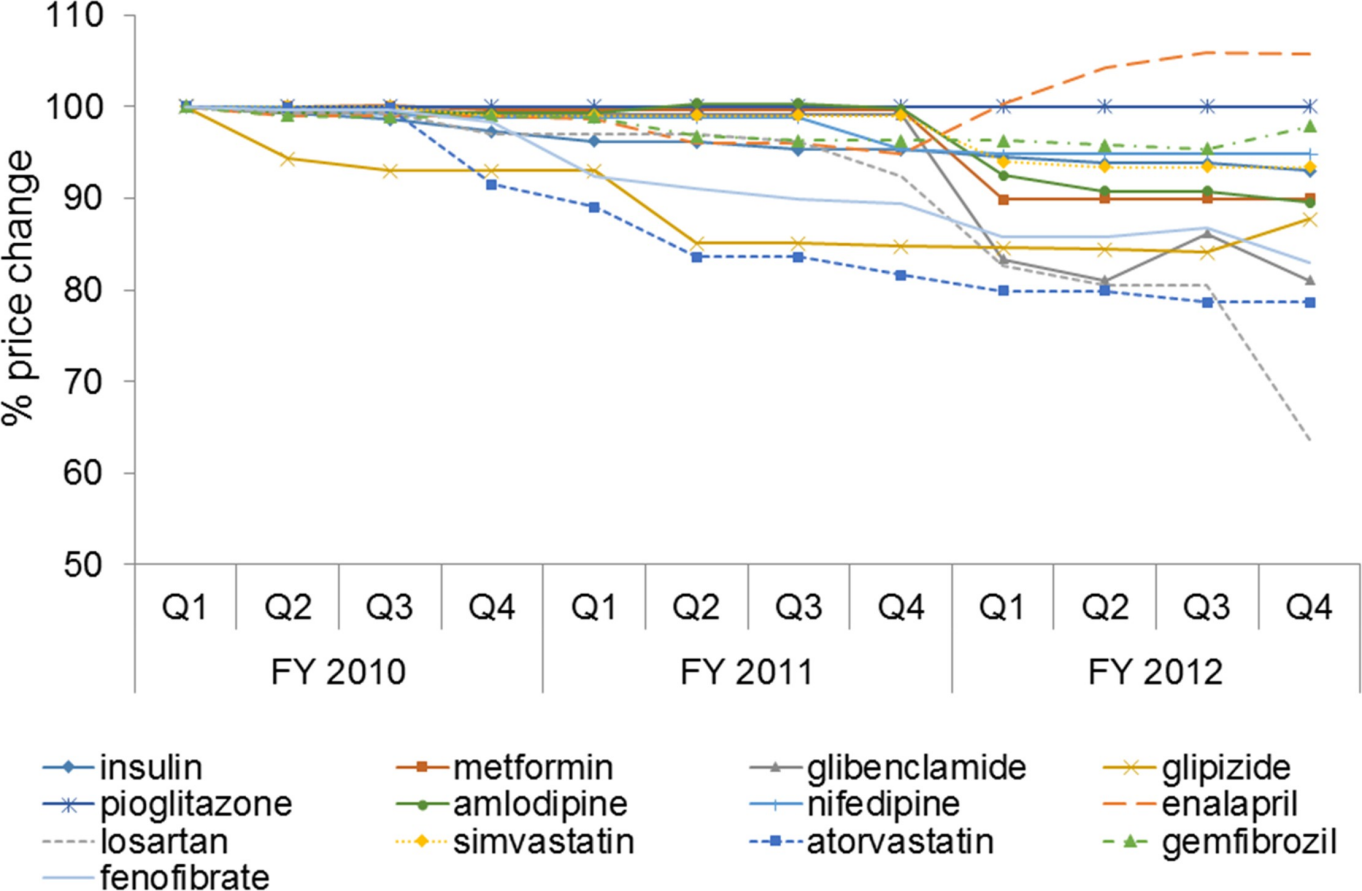
Price trends of individual antidiabetic, antihypertensive and antilipidemic drugs.

Fig 2 presents price changes relative to Q1, FY 2010, for each selected drug in each Q. Most drugs except enalapril and pioglitazone had a declining price trend to varying degrees. The prices of glipizide and atorvastatin dropped earlier in Q2 and Q4, FY 2010, respectively. The geometric mean prices in Q4, FY 2012, relative to those of Q1, FY 2010, decreased 5–10% for nifedipine, simvastatin, insulin, metformin and amlodipine and 12–36% for glipizide, fenofibrate, glibenclamide, atorvastatin and losartan.

### Price index

The 166 total drug items constituting the study basket shared 76.9 and 81.4% of the total OP drug expenditures in FY 2010 and 2012, respectively (Table 3). A total of 53 items were not included in the NLEM. Fifty of these items were single-source drugs. The highest expenditure share belonged to the combined three main ATC groups at level 1: C (cardiovascular), A (alimentary) and J (anti-infectives), shared 60.2% in FY 2010 and 67.9% in FY 2012. For an individual drug item, metformin had the highest share (4.1%), whereas erythropoietin beta had the highest price (geometric mean 9,629 Baht).

**Table 3:** Drug basket and item price index.

Main group (ATC level 1) *	All drugs				Basket drugs						
	FY 2010		FY 2012		No. of items				Top-five drugs		
	No. of items	Expenditure share (%)	No. of items	Expenditure share (%)	Total	Not in NLEM	Single source	NHSO interventions^┼^	Highest expenditure share, % (price index, 2012)	Highest price, Baht in base year (price index in %, 2012)	Change in price index ≥ 10%, price in Baht in base year (price index in %, 2012)
A	174	19.4	157	27.1	23	7	6	-	Metformin, 4.1% (90.0), human insulin, 3.0% (94.9), omeprazole, 1.4% (88.2), glibenclamide, 1.3% (83.1), glipizide, 1.2% (89.6)	Human insulin, 113.0 (94.9), rosiglitazone, 67.7 (99.5), esomeprazole, 52.9 (96.8), sitagliptin, 48.6 (98.2), lansoprazole, 42.6 (93.1)	Glibenclamide, 0.2 (83.1), omeprazole, 0.7 (88.2), alfacalcidol, 4.5 (89.0), glipizide, 0.3 (89.6), metformin, 0.5 (90.0)
B	57	6.0	58	10.4	12	2	3	3	Erythropoietin (EPO) alfa, 1.4% (91.6), aspirin (ASA), 1.2%, (103.0), clopidogrel^┼^, 0.7% (73.7), ferrous fumarate, 0.5% (103.7), EPO beta, 0.4% (100.0)	EPO beta, 9629.4 (100.0), coagulation factor VIII^┼^, 3800.3 (99.0), EPO alfa^┼^, 560.9 (91.6), enoxaparin, 234.2 (99.6), cilostazol, 25.8 (99.1)	Clopidogrel^┼^, 15.2 (73.7)
C	104	22.7	97	26.9	31	15	11	-	Simvastatin, 2.6% (93.8), amlodipine, 2.5% (91.4), enalapril, 2.3% (104.9), atorvastatin, 2.0% (81.1), rosuvastatin, 1.5% (97.5)	Ezetimibe+simvastatin, 51.4 (100.0), ezetimibe, 42.0 (98.1), rosuvastatin, 41.2 (97.5), atorvastatin, 35.8 (81.1), valsartan+amlodipine, 29.2 (100.0)	Losartan, 4.8 (77.7), doxazosin, 2.7 (80.1), atorvastatin, 35.8 (81.1), fenofibrate, 8.0 (85.9)
D	88	0.8	86	0.3	0	0	0	-			
G	59	2.4	58	1.3	6	3	3	-	Alfuzosin, 0.7% (100.0), levonorgestrel, 0.6% (100.0), tamsulosin, 0.4% (98.6), finasteride, 0.3% (64.6), raloxifene, 0.2% (98.7)	Levonorgestrel, 3417.3 (100.0), raloxifene, 56.9 (98.7), dutasteride, 47.0 (97.2), tamsulosin, 31.4 (98.6), alfuzosin, 25.7 (100.0)	Finasteride, 25.1 (64.6)
H	17	1.4	21	1.5	3	0	0	-	Propylthiouracil, 0.9% (83.7), prednisolone, 0.2% (99.2), calcitonin, 0.2% (96.4)	Calcitonin, 2537.6 (96.4), propylthiouracil, 0.5 (83.7), prednisolone, 0.3 (99.2)	Propylthiouracil, 0.5 (83.7)
J	139	18.1	133	13.9	26	2	3	13	Stavudine+lamivudine+nevirapine^┼^, 2.6% (84.7), lopinavir+ ritonavir^┼^, 2.0% (70.5), Purified Chick Embryo Cell Rabies Vaccine (PCEC), 1.6% (93.5), amoxicillin, 1.3% (96.3), purified Vero cell rabies vaccine (PVRV), 1.3% (100.0)	Human Rabies Immunoglobulin (HRIG), 2253.4 (81.9), Equine Rabies Immunoglobulin (ERIG), 709.7 (99.4), entecavir, 310.5 (98.4), PVRV, 286.8 (100.0), PCEC, 274.8 (93.5)	Efavirenz^┼^, 12.5 (58.6), lopinavir+ritonavir^┼^, 20.5 (70.5), lamivudine^┼^, 13.8 (74.3), zidovudine+lamivudine+nevirapine^┼^, 19.2 (79.7), HRIG, 2253.4 (81.9), stavudine+lamivudine+nevirapine^┼^, 13.1 (84.7), nevirapine^┼^, 9.1 (86.5), zidovudine+lamivudine^┼^, 31.0, (86.5), ofloxacin, 1.5 (87.1)
L	70	4.4	76	2.8	12	3	8	3	Imatinib^┼^, 0.9% (95.5), capecitabine, 0.7% (99.1), peginterferon alfa-2a, 0.5% (96.9), letrozole^┼^, 0.4% (100.0), paclitaxel, 0.4% (64.5)	Peginterferon alfa-2a, 9613.3 (96.9), leuprorelin^┼^, 8881.0 (99.0), paclitaxel, 6553.9 (64.5), imatinib^┼^, 1180.1 (95.5), bicalutamide, 269.2 (100.0)	Paclitaxel, 6553.9 (64.5), azathioprine, 13.9 (81.4)
M	61	5.8	62	2.0	13	9	3	-	Risedronate, 0.8% (98.6), celecoxib, 0.8% (91.9), glucosamine, 0.7% (100.0), tolperisone, 0.5% (95.8) orphenadrine+paracetamol, 0.5% (103.7)	Hyaluronate, 1764.7 (100.0), risedronate, 226.9, (98.6), diacerein, 32.4 (96.2), etoricoxib, 32.4 (98.9), celecoxib, 22.7 (91.9)	Meloxicam, 4.3 (83.2)
N	109	9.8	108	9.6	25	9	9	1	Paracetamol, 1.2% (104.1), sodium valproate, 0.8% (98.7), rivastigmine, 0.7% (100.0), phenytoin, 0.7% (136.2), perphenazine, 0.5% (99.4)	Rivastigmine, 1373.3 (100.0), olanzapine, 142.1 (92.0), donepezil, 136.6 (83.2), memantine, 81.9 (96.6), ziprasidone, 80.0 (94.0)	Risperidone^┼^, 21.3 (61.5), gabapentin, 6.2 (77.7), donepezil, 136.6 (83.2), tramadol, 0.5 (86.6), phenytoin, 1.7 (136.2)
P	19	0.2	18	0.09	0	0	0	-			
R	80	6.9	85	3.3	12	2	2	-	Salmeterol+fluticasone, 1.1% (59.8), theophylline, 0.9% (91.8), budesonide, 0.9% (101.3), salbutamol, 0.7% (91.6), ipratropium bromide+fenoterol hydrobromide, 0.7% (99.8)	Budesonide+formoterol, 1144.2 (76.7), salmeterol+fluticasone, 655.6 (59.8), budesonide, 176. 7 (101.3), salbutamol, 112.9 (91.6), ipratropium+fenoterol, 47.4 (99.8)	Salmeterol+fluticasone, 655.6 (59.8), cetirizine, 0.4 (60.6), Budesonide+formoterol, 1144.2 (76.7), loratadine, 0.6 (76.8)
S	79	1.9	73	0.6	3	1	2	-	Latanoprost, 1.0% (92.3), hypromellose, 0.6% (99.8), sodium hyaluronate, 0.4% (102.5)	Latanoprost, 458.0 (92.3), sodium hyaluronate, 265.1 (102.5), hypromellose, 36.4 (99.8)	-
V	21	0.3	21	0.3	0	0	0	-			
Total	1,082	100	1,052	100	166	53	50	20	Metformin, 4.1% (90.0), mixtard, 3.0% (94.9), stavudine+lamivudine+nevirapine^┼^, 2.6% (84.7), simvastatin, 2.6% (95.8), amlodipine, 2.5% (91.4)	EPO beta, 9629.4 (100.0), Peg-interferon alfa 2 a, 9613.3 (96.9), leuprorelin^┼^, 8881.0 (99.0), paclitaxel, 6553.9 (64.5), coagulation factor VIII^┼^, 3800.3 (99.0)	Price change >35%: efavirenz^┼^, 12.5 (58.6), salmeterol+fluticasone, 655.6 (59.8), cetirizine, 0.4 (60.6), risperidone^┼^, 21.3 (61.5), phenytoin, 1.7 (136.3), paclitaxel, 6553.9 (64.5), finasteride, 25.1 (64.6)

ATC, Anatomical, Therapeutic and Chemical Classification; FY, fiscal year; NLEM, National List of Essential Medicines; NHSO, National Health Security Office.

*A = Alimentary tract and metabolism, B = Blood and blood forming organs, C = Cardiovascular system, D = Dermatologicals, G = Genito-urinary system and sex hormones, H = Systemic hormonal preparations, J = Antiinfectives for systemic use, L = Antineoplastic and immunomodulating agents, M = Musculo-skeletal system, N = Nervous system, P = Antiparasitic products, R = Respiratory system, S = Sensory organs, V = Various.

^┼^NHSO conducted central purchasing via price negotiation by volume-based agreement.

Table 2 presents the characteristics of the drug basket and price indices in FY 2012 for the selected drug items. A total of 125 out of 166 drug items had a decreased price index in FY 2012. Sixty seven items had a less than 5% price reduction, 25 items had a 5–9% price reduction, 19 items had a 10–19% price reduction, and 14 items had at least a 20% price reduction. The prices of the most commonly used antidiabetics, including metformin, glipizide, and glibenclamide, decreased by 10, 10, and 17%, respectively, which was most likely due to the high prescription volume and market competition from generic drugs. Similarly, the prices of antihypertensive agents, including amlodipine, losartan and doxazosin, and antilipidemics, including simvastatin, atorvastatin and fenofibrate, decreased by a range of 6–22%. In addition, finasteride, gabapentin and loratadine were available with competitive prices after recent patent expiration; therefore, their prices decreased substantially (22–35%). In contrast, the price of phenytoin increased, because most hospitals switched from the generic to a branded originator product. In each ATC level 1 group, the top five drugs with the highest prices, which were relatively new and were expected to exhibit future growth, had their prices decreased minimally. Only donepezil, celecoxib, and latanoprost had their prices decreased by 8–17% during the study period.

**Fig 3:**
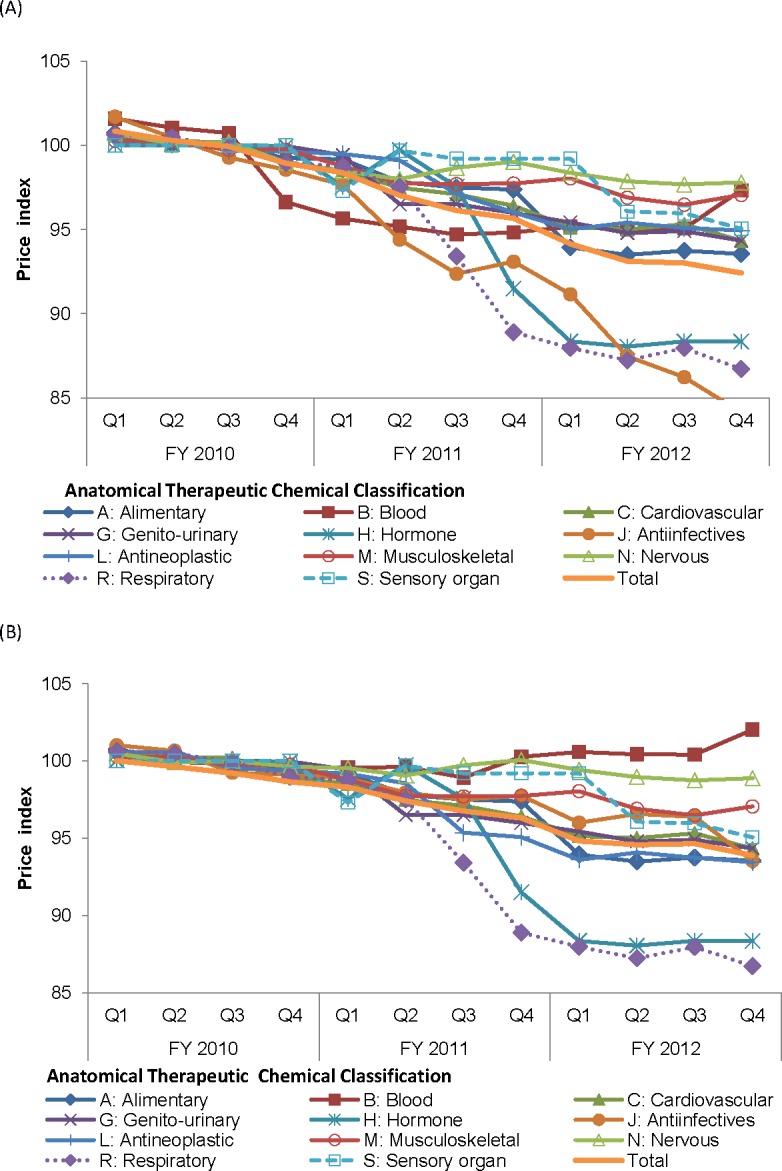
Price index. (A) All-item basket. (B) The drug basket, excluding the drugs for which the NHSO conducted price negotiations. NHSO, National Health Security Office.

The overall price index continually declined from the set average of 100% in the base year (FY 2010) to an average of 93.2% in FY 2012 (Fig 3A). The three largest price drops in FY 2012 at ATC level 1 appeared in anti-infectives (87.2%), respiratory system drugs (87.5%), and hormones (88.3%). When excluding the 20 items for which the NHSO conducted price negotiation, the overall price index dropped to an average of 95.1% in FY 2012 (Fig 3B). These 20 items contributed to 14% of the expenditure in the drug basket. Sixteen of the 20 items that the NHSO intervened with price negotiation had substantial price reductions (Table 3). Oseltamivir, letrozole, leuprolerin and coagulation factor VIII did not have price reductions. As a result, the price indices of antiretrovirals and risperidone decreased substantially by more than 10% and that of clopidogrel decreased by 26%. Combined antiretroviral products had price index decreases of 14–30%. The highest price reduction was found for efavirenz (41%) and the lowest for tenofovir (4%). The Thoracic Society of Thailand in collaboration with the NHSO promoted disease management programs for asthma and chronic obstructive pulmonary disease during the study period, which led to price reductions of expensive inhalers of 23% to 40%.

## Discussion

Our study showed a marked increase in access to essential drugs for diabetes (metformin), hypertension (enalapril and amlodipine), and dyslipidemia (simvastatin) even though their price drops were modest to moderate. Most commonly prescribed drugs that contributed relatively largely to OP expenditures and drugs with relatively high prices had a declining price trend due to generic market competition. For the all-item basket, which accounted for approximately 75% of the OP drug expenditure, the overall price index decreased by 6.8% in 2012. Price negotiation by the NHSO led to a price reduction of sixteen out of 20 items with a range from 4% to 41%. Excluding the price-negotiated items, the price index decreased by 4.9% in 2012.

Despite a limited OP budget, we assumed based on the study findings that public hospitals in Thailand were able to handle an increase in the observed demand for essential NCD medicines. One explanation was that a price drop would offset the budget required for increased access. This study shed light on the need for drug price monitoring by the UCS to assess the adequacy of the capitation budget for meeting the demand for drugs. A decrease in access coupled with an increasing price trend sends a strong signal of a need for corrective policy measures.

The upward trend in drug access was partly driven by an increase in the NCD prevalence.[6,15] Although the patients had free access to health services covered by the UCS, the growth in drug recipients was higher than that in the elderly population and patients diagnosed with diabetes and CVS diseases. The increase in the use of metformin and decrease in the use of glibenclamide and nifedipine were consistent with current clinical practice guidelines.[16–18] Awareness of the adverse effects of glibenclamide and nifedipine has been raised in most hospitals and has resulted in a decrease in their utilization.

The most recent (2014) National Health Examination Survey (NHES) of the population aged 15 years or over in Thailand, which measured the fasting plasma glucose (FPG), blood pressure (BP) and total cholesterol (TC) levels on the survey dates, revealed prevalence rates for diabetes (FPG ≥ 126 mg/dl), hypertension (BP ≥ 140/90 mmHg) and dyslipidemia (TC after fasting ≥ 240 mg/dl) of 8.9%, 24.7% and 16.4% respectively.[15] Nearly half of the surveyed adults with diabetes (43.2%) and hypertension (44.7%), and 61.7% of those with dyslipidemia had not been previously diagnosed. Of the diagnosed patients, 95.2% received treatments for diabetes, 89.0% for hypertension, and 84.6% for dyslipidemia. Of the treated adults, 60.4% were in controlled conditions for hypertension, 43.4% for diabetes, and 85.5% for dyslipidemia. Of the NHES-based adult population, 4.8% received treatments for diabetes, 12.2% for hypertension, and 5.3% for dyslipidemia. Our study found that at Q4, FY 2012, access to the studied antidiabetics and antilipidemics was limited to 30 and 47 per 1,000 population, respectively. The number of patients receiving antihypertensive agents of approximately 50 per 1,000 population was lower than that of the survey. Access to the antihypertensive agents in our analysis did not account for hydrochlorothiazide, which was known to be used widely in patients with hypertension. Our supplemental analysis (data not shown) found that 118 per 1,000 UCS population received any antihypertensive agent. Taking into account the estimated quarterly growth, access to the four antidiabetics in our study was slightly lower than the national figures.

Update information on drug purchasing prices is necessary to plan for the OP budget, because drug use is a major component of OP services. The WHO has recommended using the price index as an indicator for monitoring the affordability of medicines.[13] Countries that report the drug price index regularly include Canada and the USA.[19,20] Our study used hospital OP and drug purchasing databases that had potential as routine data sources.

The major limitations of this study were as follows. For drug access, a drug recipient indicated for medical treatment is the ideal numerator. However, an intensive chart review to identify patients with a medical need is a time-consuming approach with a prohibitive cost. Access to medicines is complex, with a notable lack of monitoring data. Proxy indicators are commonly used, such as the utilization rate based on the ratio of the population to the number of drug recipients, DDDs, sales value, or use volume.[21,22] In our study, the increasing trend in access based on the population was consistent with the increasing trend in the prevalence of diabetics, hypertension and dyslipidemia obtained from both national surveys and facility-based data.[6,15]

For the price index, rebasing and reweighting the drug basket are issues of concerns. A typical basket tended to include few newly launched, high-cost products due to their small initial market share. Our study handled this issue by incorporating key new products expected to have growth in the near future. The chain-base index applied in our study is sensitive to technological change; however, our study relied on a relatively short period that was not sufficient for rebasing and reweighing. The drug items included in our study were selected from drugs that were commonly used in hospital OP settings, and the base-year (FY 2010) basket was found to be a good representative up to FY 2012.

Further studies are needed to gain an in-depth understanding of the population that continues to have inaccessibility issues for essential drugs. Although the UCS patients were not required to pay for the essential drugs and all healthcare deemed essential, non-healthcare costs borne by the patients, such as transportation and lost earnings, need to be acknowledged as remaining barriers to access to care. Furthermore, intangible behavioral factors, including attitude and awareness, play an important role on the demand side in achieving treatment goals.

In summary, access to essential drugs for diabetes, hypertension and dyslipidemia has increased over time. The decline in the overall price index of 6.8% during the three years of the study period could help hospitals mitigate the total costs due to increasing access. Market competition of generic drugs and price negotiation by the NHSO for UCS led to the reduction in drug prices.

## Supporting information

S1 Table Ages of the drug recipients and numbers of drug items per visit, FY 2010–2012. (DOCX)

S1 File Price index calculation. (DOC)
