# Supplementary material for: Access to and price trends of antidiabetic, antihypertensive, and antilipidemic drugs in outpatient settings of the Universal Coverage Scheme in Thailand
Source: PLoS One. 2019 Feb 20;14(2):e0211759. doi: 10.1371/journal.pone.0211759 (PMC6382105; doi:10.1371/journal.pone.0211759)
Supplement: S1 Table — (DOCX) [file pone.0211759.s001.docx]

**S1 Table. Ages of the drug recipients and numbers of drug items per visit, FY 2010-2012**

|  | FY 2010 | FY 2011 | FY 2012 |
| --- | --- | --- | --- |
| Ages of drug recipients in years, mean (SD) | | | |
| Antidiabetics | 59.3 (13.1) | 59.4 (13.7) | 59.6 (14.6) |
| Antihypertensive agents | 62.0 (19.1) | 62.2 (17.9) | 62.4 (14.3) |
| Antihyperlipidemics | 61.1 (14.9) | 61.1 (15.3) | 61.4 (14.7) |
| Adult recipients (15-59 years) | | | |
| Antidiabetics | 49.9% | 49.5% | 48.6% |
| Antihypertensive agents | 41.0% | 40.8% | 40.4% |
| Antihyperlipidemics | 44.7% | 44.8% | 43.9% |
| Elderly recipients (> 60 years) | | | |
| Antidiabetics | 49.8% | 50.3% | 51.3% |
| Antihypertensive agents | 58.7% | 59.0% | 59.4% |
| Antihyperlipidemics | 55.1% | 55.1% | 56.0% |
| Number of drug items per visit, mean (SD) | | | |
| Antidiabetics, oral | 1.61 (0.55) | 1.63 (0.56) | 1.64 (0.57) |
| Antihypertensive agents | 1.21 (0.41) | 1.23 (0.42) | 1.26 (0.44) |
| Antihyperlipidemics | 1.04 (0.17) | 1.04 (0.16) | 1.04 (0.15) |
