# Supplementary material for: Access to and price trends of antidiabetic, antihypertensive, and antilipidemic drugs in outpatient settings of the Universal Coverage Scheme in Thailand
Source: PLoS One. 2019 Feb 20;14(2):e0211759. doi: 10.1371/journal.pone.0211759 (PMC6382105; doi:10.1371/journal.pone.0211759)
Supplement: S1 File — (DOC) [file pone.0211759.s002.doc]

# S1 Price index calculation

For an ATC level 5 drug, the unit prices in each quarter across dosage strength-forms and hospital markets were averaged using a geometric mean (see equation 1) to reduce influence from an extremity of price dispersion.

……………………………. (1)

For an individual drug *i* at ATC level 5, the geometric mean price in quarter *j* (*p_ij_*) from equation 1 was compared with the first four-quarter average of the geometric mean in the base year (*p_i_*_0_) as a ratio and then weighted by relative contribution (in percentages) to the whole basket expenditure (*w_i_*) (equation 2) to obtain the weighted price ratio (*w_ij_*).

…………………………………. (2)

For each of the first four quarters in the base year, the price index for ATC level 5 (*I_ij_*) was calculated by dividing the weighted price ratio (*w_ij_*) from equation 2 by the percent expenditure contribution (*w_i_*) as in equation 3, which in turn equaled the ratio between the quarterly price (*p_ij_*) and the base-year price (*p_i_*_0_).

$I_{ij}=\frac{w_{ij}}{w_{i}}=\frac{p_{ij}}{p_{i0}}$ …………………………………. (3)

For the fifth quarter onwards, the price index in each quarter was calculated using the Lowe index or modified Laspeyres index formula, which were chain-base methods (equation 4). For example, *I_i_*_5_ is equal to a ratio between *w_i_*_5_ and *w_i_*_4_ and then is multiplied by *I_i_*_4_, and so on.

$I_{ij}=\frac{w_{ij}}{w_{ij-1}}\times I_{ij-1}$ …………………………………. (4)

For the price index of a higher aggregate, anatomical codes are grouped by ATC level 1. For every quarter, the weighted price ratios of the level 5 ATC drugs (*w_ij_*) from equation 2 were summed over the level 1 ATC group (*w_j_*) (equation 5).

{\displaystyle t} $w_{j}=\sum_{i} \left[w_{i}\frac{p_{ij}}{p_{i0}}\right]$ …………………………………. (5)

To generate the price index for the aggregate ATC level 1 group in each of the first four quarters, the price index was the ratio between the summed weighted ratios across ATC level 5 drugs and the summed percent expenditure contribution (*w_i_*).

$I_{j}=\frac{w_{j}}{\sum_{i} w_{i}}$ …………………………………. (6)

For the price index for the fifth quarter onwards, the index in a current quarter (*j*) was chained to the index from a previous adjacent quarter (*j*-1), as in equation 7.

$I_{j}=\frac{w_{j}}{w_{j-1}}\times I_{j-1}$ …………………………………. (7)

Calculation of the price index for the highest level (i.e., the whole drug basket) was repeated similar to equations 6 and 7. For the base-year quarter, the price index was equal to the summation of the weighted price ratios across the ATC level 1 groups. Then, the price index of the fifth quarter onwards was chained to the fourth quarter index, and so on.
